# Multislice CT-guided evaluation of collagen–chitosan composite in promoting antebrachiocarpal arthrodesis in a rabbit model

**DOI:** 10.1186/s13620-025-00307-1

**Published:** 2025-08-26

**Authors:** Gannah-Samy, Alaa Samy, Awad Rizk, Emad Tolba, Zainab A. Ramadan, Gamal Karrouf

**Affiliations:** 1 Department of Surgery, Anesthesiology, and Radiology, Faculty of Veterinary Medicine, University of Mansoura, Mansoura, 35516 Egypt; 2 Polymers and Pigments Department, National Research Centre, 33 El Bohouth St, P.O. 12622, Dokki, Giza Egypt; 3 Department of Diagnostic and Interventional Radiology, Faculty of Medicine, Mansoura University, Mansoura, 35516 Egypt; 4 Department of Surgery, Anesthesiology, and Radiology, Faculty of Veterinary Medicine, Mansoura University, Mansoura, 35516 Egypt

**Keywords:** Collagen-chitosan, Bone fusion, Antebrachiocarpal, Arthrodesis

## Abstract

**Background:**

Arthrodesis is a critical procedure for restoring stability and relieving pain in severely damaged joints. Successful bone fusion remains a significant challenge, often necessitating the use of biomaterials to enhance healing. Collagen and chitosan, two natural polymers with established biocompatibility and osteoconductive properties, have shown promise in regenerative medicine applications. The present study aimed to evaluate the synergistic effect of a collagen-chitosan composite on bone fusion of the antebrachiocarpal joint in a rabbit model. Multislice CT morphometrical analysis was utilized to assess bone healing and fusion, offering detailed insights into the material’s efficacy in promoting joint stabilization and bone regeneration.

**Materials and methods:**

Twelve healthy male New Zealand White rabbits (4.0 ± 0.3 months old) with a mean body weight of 2.5 ± 0.5 kg were used. These animals underwent curettage of the articular cartilage down to the subchondral bone. The rabbits were then randomly assigned into two groups: a control group (C), in which no composite was applied, and a treatment group, in which collagen-chitosan scaffolds were utilized (Col/Cs). Joint fusion was postoperatively assessed using a multislice detector computed tomography (MSCT).

**Result:**

(MSCT) revealed progressive enhancements in the collagen–chitosan (Col/Cs) group over 12 weeks. Radial cortical thickness and bone mineral density (BMD) were significantly higher at week 12 in the Col/Cs group (1.31 ± 0.10 mm vs. 1.03 ± 0.18 mm; *p* = 0.0086, and ~ 760 HU vs. ~510 HU; *p* = 0.0055, respectively). Intra-articular mineral density (IATMD) increased markedly at week 1 (*p* < 0.0001), decreased at week 6 (*p* < 0.0001), and rose again by week 12 (*p* < 0.0001), while the control group showed a gradual, non-significant increase. Joint space width decreased significantly in the Col/Cs group by week 6 (~ 0.6 mm vs. ~0.9 mm; *p* = 0.0034) and remained lower at week 12 (~ 0.55 mm vs. ~0.7 mm; *p* = 0.0062). Fusion ratio reached ~ 65% in the Col/Cs group compared to ~ 35% in controls (*p* < 0.0001). CBMD decreased in both groups by week 1 postoperatively but recovered more effectively in the Col/Cs group. By week 12, CBMD was significantly higher in the Col/Cs group (~ 1000 HU) than in controls (~ 950 HU; *P* < 0.0006). (UBMD) was initially similar (~ 780 HU), but by week 1, the Col/Cs group maintained higher values (~ 760 HU vs. ~620 HU; *p* < 0.0001), and this inclination continued through week 12 (~ 750 HU vs. ~680 HU; *p* = 0.001).

**Conclusion:**

The results of the present study indicate that the collagen–chitosan composite enhances bone fusion and joint stability in a rabbit model of antebrachiocarpal arthrodesis, demonstrating both innovation and potential clinical applicability.

## Introduction

Arthrodesis is considered when non-surgical treatments are no longer effective. The primary objectives of the procedure are to alleviate pain and enhance the patient’s functional ability [1]. Various techniques have been developed over the years to achieve successful arthrodesis in both human and veterinary orthopedic applications. Traditional methods often rely on the curettage of articular cartilage and application of metallic fixation devices such as plates and screws [2]. Arthrodesis of the carpal joints, whether the antebrachiocarpal joint, the middle carpal/carpometacarpal joint, or the entirety of the carpus, powerfully enables weight-bearing without pain, leading to a profound restoration of function [3].

The persistently high nonunion rates continue to pose a major challenge necessitating the exploration of tissue engineering and regenerative medicine to achieve effective bone augmentation and full regeneration [4]. These outcomes remain unattainable without the incorporation of osteogenic or osteoinductive materials to stimulate bone formation [5].Biomaterials play a crucial role in bone regeneration by providing structural support and creating a favorable environment for new bone growth [6–8].

Natural polymers, such as collagen, chitosan, gelatin, silk fibroin, alginate, and cellulose, are increasingly utilized in bone tissue engineering and arthrodesis due to their excellent biocompatibility, biodegradability, and ability to mimic the natural extracellular matrix [6]. Chitosan (CS) has been widely utilized in bone tissue engineering due to its ability to promote osteoblast growth and the deposition of a mineral-rich matrix in culture [7]. It is also adept at delivering vital growth factors, including platelet-derived growth factor (PDGF) and bone morphogenetic protein 2 (BMP-2), significantly enhancing the process of bone regeneration [8]. Marine collagen, derived from sources such as fish and jellyfish, is gaining attention as a biomaterial for bone regeneration due to its biocompatibility, low immunogenicity, and sustainability compared to mammalian collagen [9]. Research highlights its ability to promote osteoblast maturation, increase mineral deposition, and density [10]. Marine collagen offers a lower risk of disease transmission and fewer ethical or religious concerns compared to bovine or porcine sources [11].

Multi-slice scan detector, commonly referred to as a multi-slice or multi-detector computed tomography (MSCT) system, uses multiple rows of detectors to simultaneously capture data from several slices of the body during a single rotation, greatly increasing scanning speed and coverage compared to single-slice CT [12]. Studies show that MSCT provides highly accurate measurements of bone density, with excellent correlation to gold-standard micro-CT assessments of bone volume fraction, making it valuable for pre-surgical planning, such as dental implant placement and other bone-related procedures [13] Although the regenerative potential (Col/Cs) was addressed in bone defect repair [14]but to the best of our knowledge, this is the first study evaluates the efficacy of a collagen-chitosan bio-compositein the context of antebrachiocarpal arthrodesis in a rabbit model. Moreover, the quantitative assessment of bone fusion and mineralization using multislice computed tomography (CT) morphometry has not been previously documented in the literature for arthrodesis evaluation.

## Materials and methods

### Animals and housing

Twelve healthy male New Zealand White rabbits (4.0 ± 0.3 months old, 2.5 ± 0.5 kg) were acclimated for two weeks before the commencement of the study. The animals were housed under controlled conditions at the Surgery Department, Mansoura Veterinary Teaching Hospital, Mansoura University, Egypt, with a stable temperature of 22° ± 1 °C, 55% humidity, and a 12-hour light/dark cycle. Throughout the study, the rabbits had ad libitum access to a standard diet and fresh water. All procedures involving animal use and handling adhered to the *Guide for the Care and Use of Laboratory Animals* and were approved by the Faculty of Veterinary Medicine Ethics Committee, Mansoura University, Egypt (registration code: MUACUC (VM.MS.24.04.132)). Additionally, the study followed the ARRIVE guidelines to ensure ethical and scientifically rigorous research practices.

### Experimental design

Twelve rabbits underwent curettage of the articular cartilage down to the subchondral bone. These animals were then randomly assigned into two groups: a control group (C), in which no scaffold was applied, and a treatment group, in which collagen-chitosan scaffolds were utilized (Col/Cs).

### Preparation and microstructure characterization of collagen/chitosan (Col/Cs) composite scaffolds

Hydrolyzed Deep Sea Marine Collagen (Col) peptides were purchased from Beijing SEMNL Biotechnology (China). Chitosan (CS) with MW 1 × 10^12^ g/mol was purchased from Sigma Aldrich (Germany).

The collagen/chitosan hydrogels were prepared by freeze-gelation and freeze-drying methods. In brief, 2 g of chitosan was dissolved in 50 mL of acetic acid aqueous solution (typically 1% v/v acetic acid). The chitosan solution was kept under vigorous stirring for 1 h at room temperature. In parallel, 2 g of marine collagen was dissolved in 50 mL of distilled water at 37 °C for 10 min. Then, equal volumes of chitosan and collagen solutions were mixed and kept under slow stirring for 1 h. Then, the Col/Cs solution was homogenized at 6000 rpm for 10 min using a high-shear homogenizer, followed by magnetic stirring at 600 rpm for 6 h. The obtained Col/CS solutions were poured into sterile plastic petri dishes with Lids [90 mm (diameter) x 15 mm (depth)] and frozen at −20 °C for 24 h. The frozen dishes were immersed in a NaOH/ethanol aqueous solution and kept at −20 °C overnight. After that, the hydrogels were gently removed from plastic petri dishes and washed with ethanol solution to remove excess NaOH solution and precooled to −20 °C for another 24 h. Finally, the hydrogels were lyophilized at −56 °C to remove the aqueous solution contained in the frozen hydrogels.

The COL/Cs scaffold samples were coated with gold and examined using a field emission scanning electron microscope (SEM) (Jeol JXA 840, Japan).

### Surgical procedure

A preoperative antibiotic, Cefotaxime sodium, was injected intravenously at a dose of 50 mg/kg (Cefotax, EIPICO Pharmaceutical Company, Egypt). General anesthesia was induced with 7.5 mg/kg xylazine (Xylaject, 20 mg/ml; Adwia Co., Egypt) and 40 mg/kg ketamine hydrochloride (Ketamine 50 mg/10 ml, Rotexmedica, Germany). Under complete aseptic conditions and in lateral recumbency, a 3 cm longitudinal incision was made over the dorsal aspect of the right antebrachium, starting from the lower third of the radius and extending toward the carpometacarpal joint. The subcutaneous tissues and antebrachial fascia were carefully dissected, taking special care to avoid damaging the cephalic vein. The two branches of the cephalic vein were identified, dissected, and isolated laterally from the antebrachiocarpal joint. The common digital extensor tendon was located and retracted laterally. Using a No. 10 blade, an incision was made to open the joint capsule. The upper and lower articular cartilages were meticulously curetted down to the subchondral bone with a No. 10 blade and a small bone curette, ensuring that bleeding was evident. The joint space was thoroughly irrigated with normal saline. In the control group, no biomaterial was applied, while in the Col/Cs group, a 0.5 × 0.25 cm scaffold was placed into the joint space, ensuring direct contact with the exposed bone surfaces. The joint capsule and the subcutaneous tissue were sutured individually using a 5/0 Maxon suture (Covidien 8886621321, 1 × 30”, CV-22/T-30, green) in a simple continuous pattern, followed by an intra-articular injection of Gentamycin (20 mg/2 ml). The skin was routinely closed, and a full limb cast was applied using Optima Cast (4-inch, Blue Orthopedic Casting Tape, 10 cm × 3.6 m).

### Postoperative care

Preoperative antibiotic was continued for 5 days, and meloxicam (Anticox II 15 mg, ADWIA, Egypt) was administered at 0.6 mg/kg daily for 5 days.

### Multislice CT scanning and interpretation

Multislice CT quantitative and qualitative joint fusion criteria have been estimated to build a picture for the progression of ankylosis up to 90 days in vivo PO period. It was performed on the 7th, 45th, and 90th PO days using GE Healthcare 128-slice CT (Revolution EVO, Waukesha, WI, USA). Protocols were adjusted with the following parameters: 100–120 kVp, 150–250 mAs (depending on automatic exposure modulation), 25–50 cm window width, large body field of view, 0.625 mm slice thickness, 0.625 mm interval (gap), and 0.984: 1 rot ~ 40 mm/s pitch & speed (helical acquisition only). Images were reconstructed using bone and soft tissue kernels.

### Image analysis

All radiographic interpretations were performed by the same experienced radiologist to avoid the influence of interpretation variables on the results. All measurements were taken from multiple slices, and the mean was calculated.

**Fig. 1:**
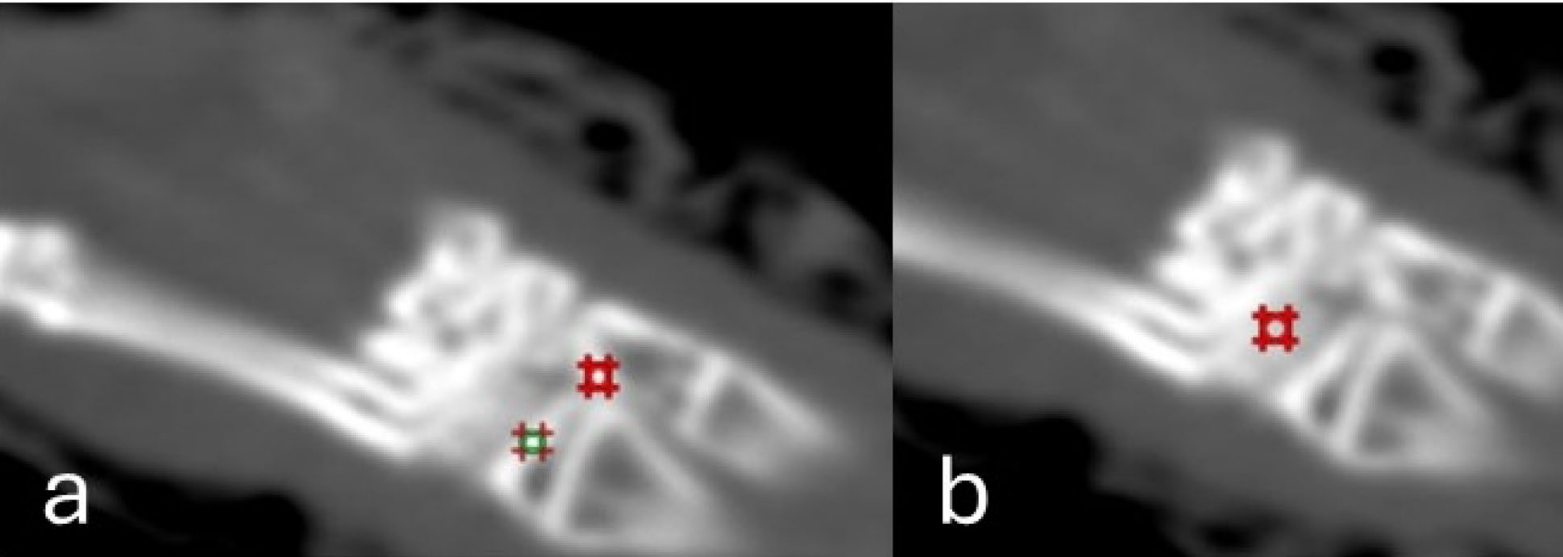
Elliptical regions of interest (ROI) drawn to measure cortical bone Hounsfield unit (HU) density at the distal end radius and ulna (**a**) and carpal bone Hounsfield unit (HU) density (**b**) at the radiocarpal joint

Radial cortical thickness (RCT) was measured in millimeters (mm) at predefined points in the middle of the distal extremity using a digital caliper within the CT workstation. Radial bone mineral density (RBMD) was measured in Hounsfield units (HU) at the distal end of the radius, where the region of interest (the point where the RBMD is measured: ROI) was standardized at approximately 5 mm² and located at the junction of the cortical bone and the adjacent medulla. The ulnar and the carpal bones, radial and ulnar BMD, were measured similarly **(**Fig. 1**).**

In the intra-articular region, ROI placement varied based on stage: on the 7th PO Day, the ROI was positioned within the joint space to measure intra-articular changes, while in the advanced weeks, it was placed in the residual joint space.

Concerning the fusion ratio, it was calculated from multiple CT slices by the following formula:$\:\:[Fusion\:ratio=\frac{\sum\:widths\:of\:\:fused\:segments}{\sum\:Width\:\:of\:joint\:surface\:}\times\:100]$ and the mean was calculated. Fusion grades were modified after Mehlhorn et al. (2020) in Table 1.

**Table 1:** Clinical assessment scores for fusion ratio modified according to Mehlhorn et al. (2020)

Score	Definition
Fusion grades	
Failed	Failed fusion was considered when fusion occurred in less than 33%.
Partial	Partial arthrodesis was defined as bone bridging, but not obliteration of the joint Focal partial fusion: associated with poor satisfaction and high risk of failure Segmented partial fusion: associated with moderate satisfaction Range of fusion from 34%−65%
Complete Sub-optimal	Range of fusion from 70–80%, clinically accepted with good satisfaction
Complete optimal	More than 80% osseous bridging across the joint on multiple slices on imaging was required to consider a successful fusion. Which is associated with excellent satisfaction

### Statistical analysis

Statistical analysis was performed using GraphPad Prism software version 8.3.4 (GraphPad Software, San Diego, CA, USA). Data were expressed as mean ± standard deviation (SD). Differences between the control and collagen-chitosan (Col/Cs) groups across multiple time points were analyzed using two-way analysis of variance (ANOVA) to assess both treatment effects and temporal changes over the 12-week postoperative period. Data normality was verified using the Shapiro–Wilk test. A p-value of less than 0.05 was considered statistically significant.

## Results

**Fig. 2:**
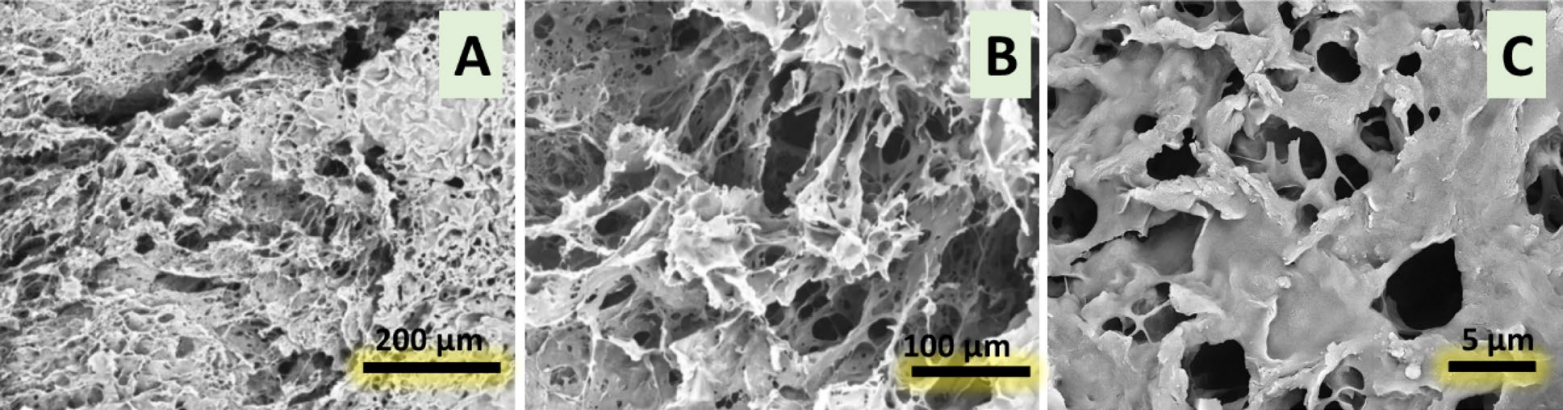
SEM analysis of Col/Cs scaffolds (JEOL JXA-840, Japan) revealed a highly porous, interconnected structure. Panel A (200 μm) showed the general pore network, Panel B (100 μm) detailed fiber pore heterogeneity, and Panel C (5 μm) illustrated fine fiber morphology and uniform pore distribution

Scanning electron microscopy of the cross-section freeze-dried samples of COL/Cs composite revealed a highly porous, interconnected structure. The overall pore network (200 μm), detailed fiber pore heterogeneity (100 μm), fine fiber morphology, and uniform pore distribution (5 μm) as shown in Fig. 2.

**Fig. 3:**
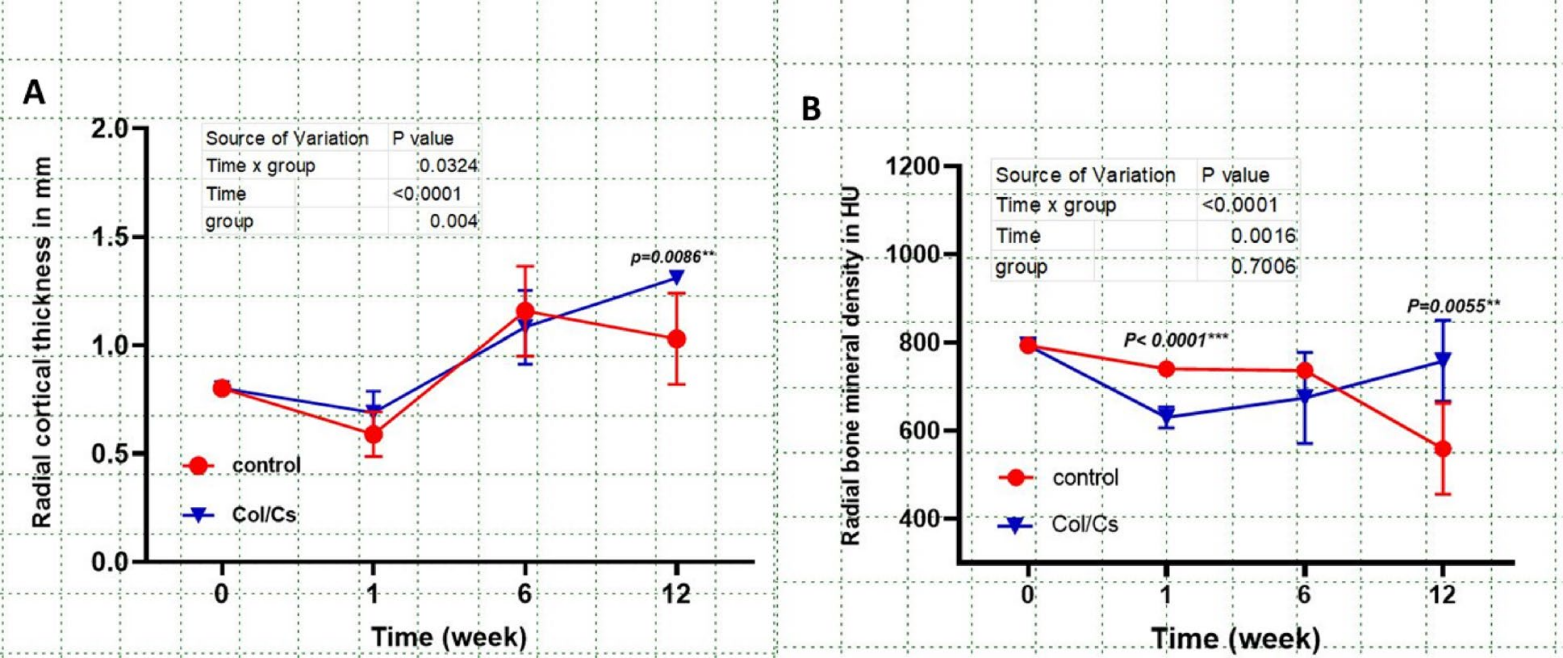
Statistical graphs illustrate radial cortical thickness RCT (**A**), radial bone mineral density RBMD (**B**). Data are expressed as mean ± SD, no of animals (*n* = 6 per group)

Quantitative analysis of radial cortical thickness (RCT) over the 12-week postoperative period demonstrated an initial decline in both groups at week 1. However, a significant time-dependent increase was observed in the Col/Cs group from week 1 to week 12 (*p* < 0.05), in contrast to the control group, which exhibited a non-significant decrease between weeks 6 and 12. At the 12-week endpoint, the Col/Cs group showed significantly greater RCT (mean ± SD: 1.31 ± 0.10 mm) compared to the control group (1.03 ± 0.18 mm; *p* = 0.0086), Fig. 3A.

Longitudinal analysis of radial bone mineral density (BMD), expressed in Hounsfield Units (HU), demonstrated a statistically significant interaction between time and treatment group. At baseline (week 0), mean BMD values were comparable between the Col/Cs and control groups (~ 780 HU). By week 1, a marked reduction was observed in the Col/Cs group (mean ± SD: ~630 HU), while the control group retained higher BMD levels (mean ± SD: ~760 HU), yielding a highly significant difference (*p* < 0.0001). Over the subsequent weeks, the Col/Cs group exhibited a progressive increase in BMD, in contrast to a gradual decline in the control group. At week 12, the Col/Cs group recorded significantly higher BMD values (mean ± SD: ~760 HU) compared to the control group (mean ± SD: ~510 HU; *p* = 0.0055), Fig. 3B.

**Fig. 4:**
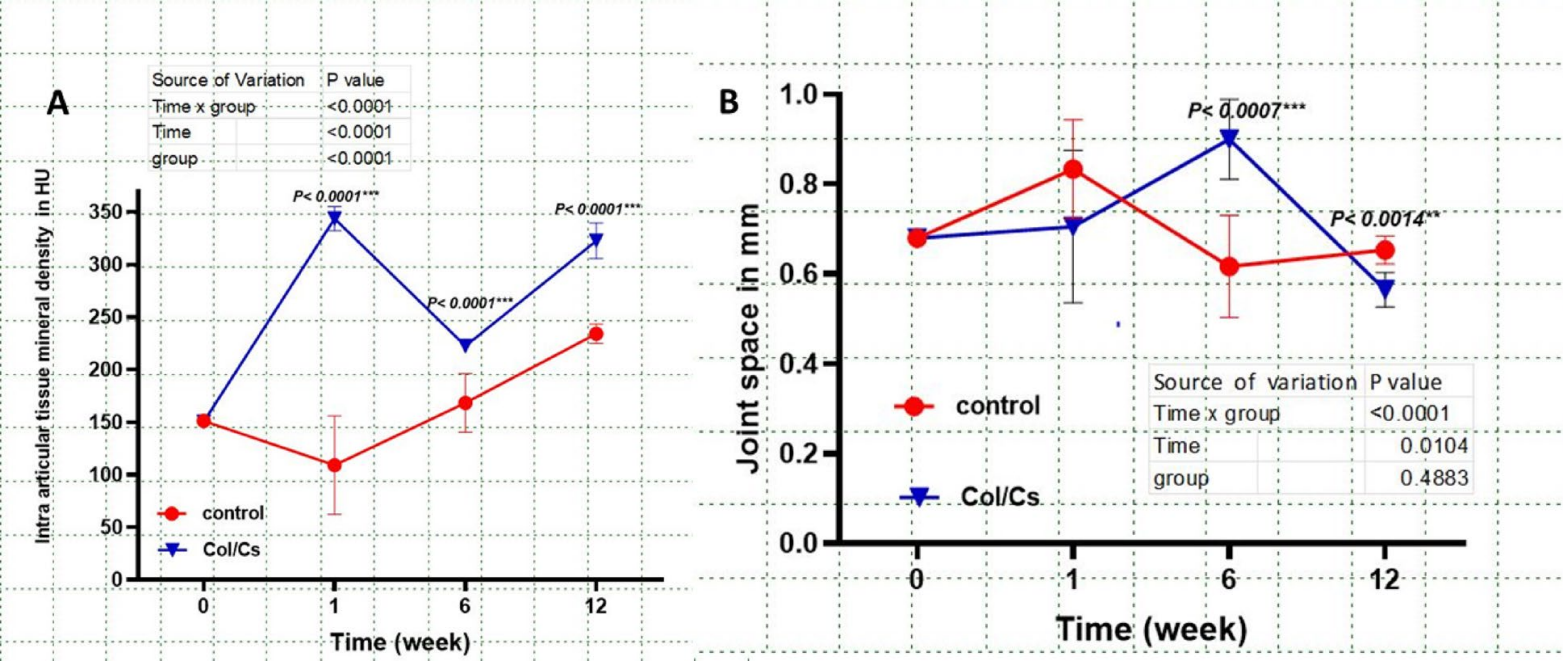
Statistical graphs illustrate intra-articular tissue mineral density IATMD (A), Joint Space(B). Data are expressed as mean ± SD, no of animals (*n* = 6 per group)

Intra-articular tissue mineral density (IATMD) demonstrated significant time and treatment-dependent variations between the control and collagen–chitosan (Col/Cs) scaffold groups over the 12-week observation period. In the Col/Cs group, a statistically significant increase was detected at week 1 compared to baseline (*p* < 0.0001), followed by a notable decline at week 6 (*p* < 0.0001), and a subsequent rebound by week 12 (*p* < 0.0001). Conversely, the control group showed a gradual upward trend in mineral density over time; however, these changes did not reach statistical significance at any time point. (Fig. 4.**A).**

Joint space evaluation revealed that both groups started with comparable baseline values (~ 0.7 mm). By week 6, the Col/Cs group exhibited a significant narrowing of the joint space (mean ± SD: ~0.6 mm), in contrast to a wider joint space in the control group (mean ± SD: ~0.9 mm; *p* = 0.0034). This trend continued through week 12, where the Col/Cs group maintained a significantly reduced joint space (mean ± SD: ~0.55 mm) relative to the control group (mean ± SD: ~0.7 mm; *p* = 0.0062). (Fig. 4B**).**

**Fig. 5:**
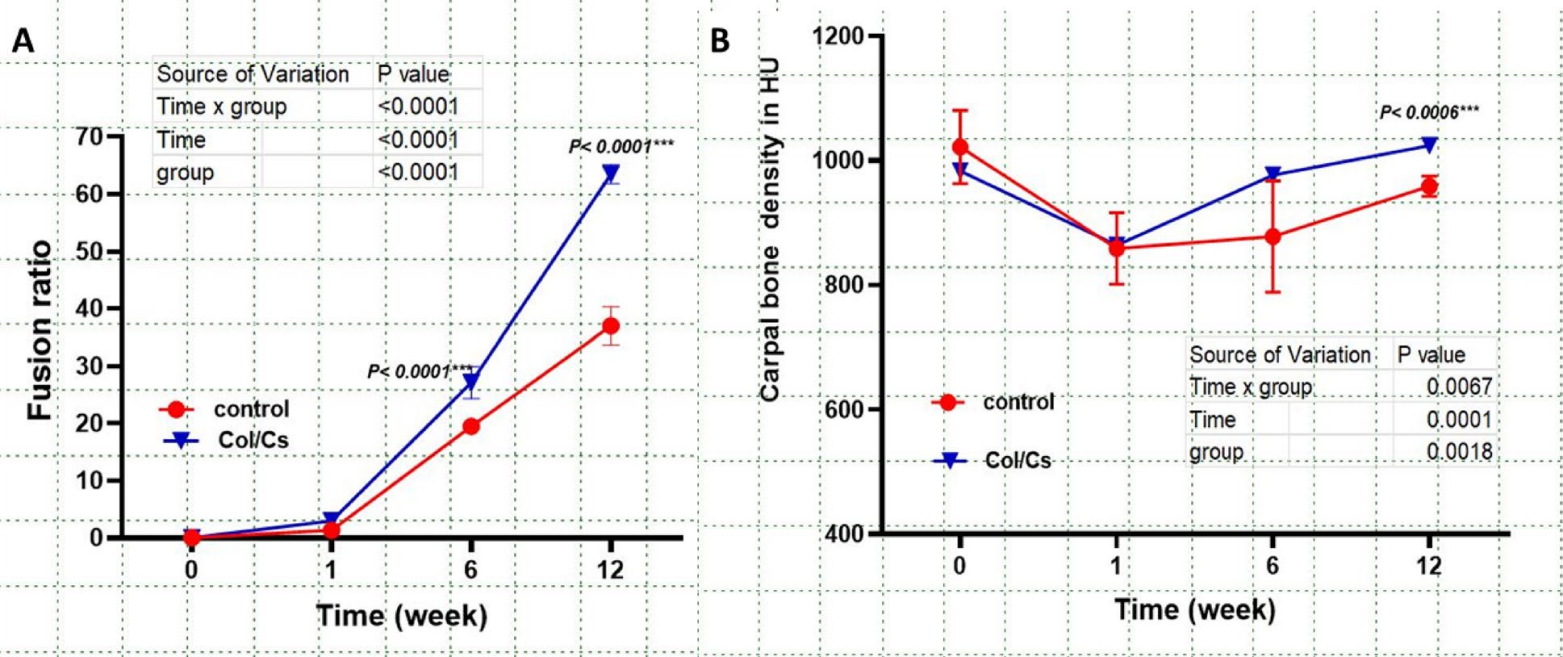
Statistical graphs show Fusion ratio (**A**), Carpal bone mineral density CBMD (B), Data are expressed as mean ± SD, no of animals (*n* = 6 per group)

**Fig. 6:**
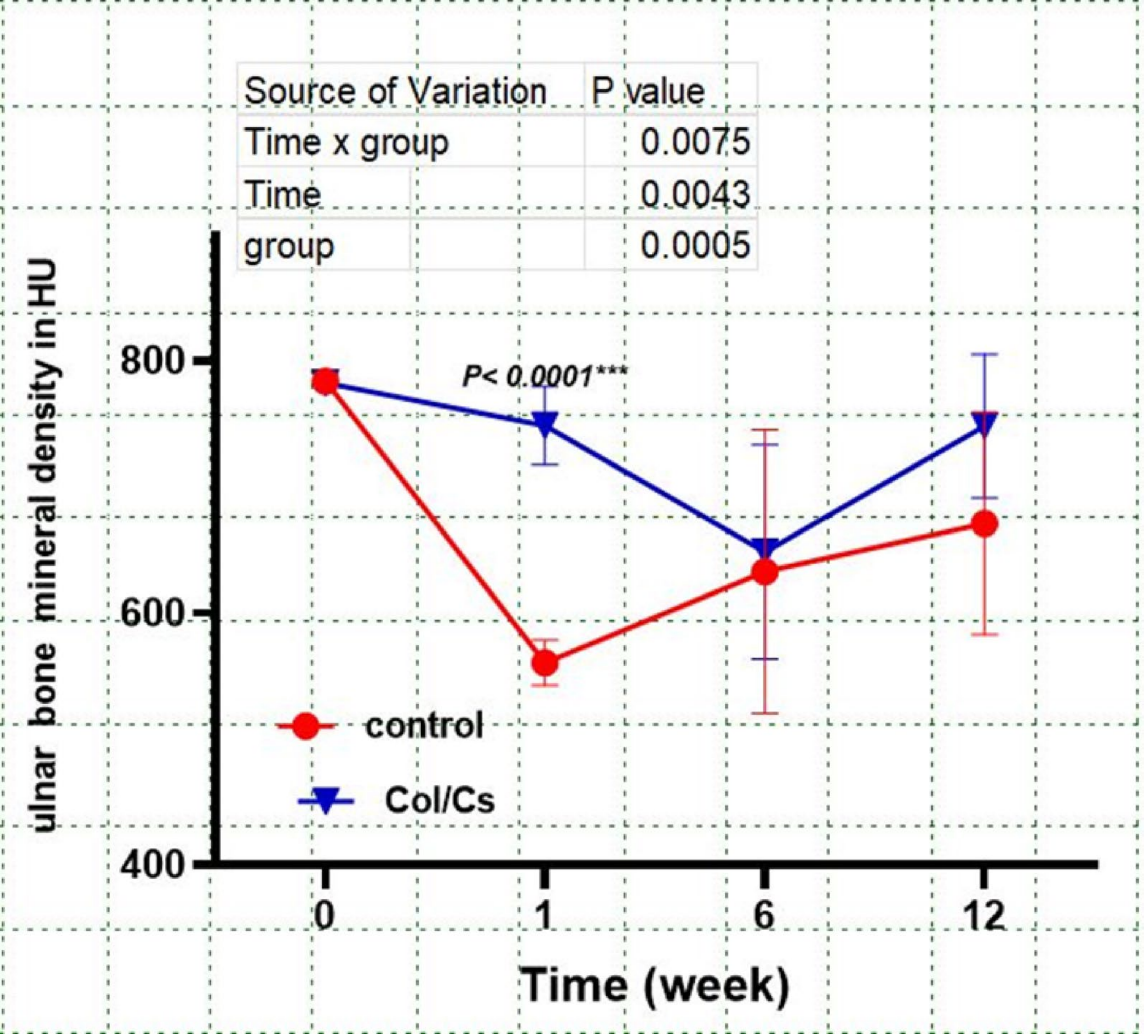
A Statistical graph demonstrates the Ulnar bone mineral density UBMD. Data are expressed as mean ± SD, no of animals (*n* = 6 per group)

**Fig. 7:**
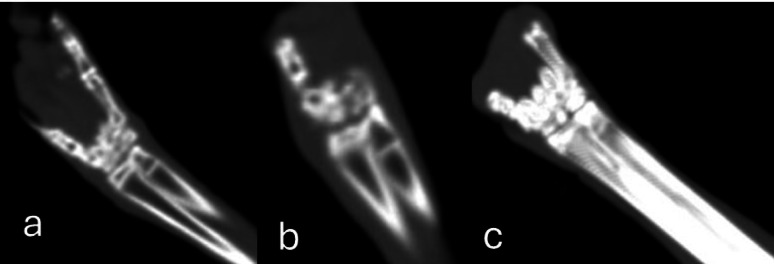
Coronal oblique reformatted CT images of the radio-carpal joint for collagen-chitosan group after intervention (a) after one week, (b) after 1.5 months, and (c) 3 months intervals showing progressive narrowing of the joint space, increased bone density, incomplete osseous bridging and fusion ratio about 39.5% and 70% at 1.5- and 3-months intervals respectively

Fusion ratio analysis revealed a significantly higher rate of bone fusion in the collagen–chitosan (Col/Cs) group compared to the control group over the 12-week postoperative period. At week 6, the Col/Cs group achieved a fusion ratio of approximately 30%, which was significantly greater than that of the control group (*p* < 0.0001). This difference became more pronounced by week 12, with the Col/Cs group reaching a fusion ratio of nearly 65%, while the control group attained only about 35% (*p* < 0.0001) as detected in (Figs. 5A, 6 and 7).

The carpal bone mineral density (CBMD) exhibited distinct temporal trends between the control and Col/Cs (collagen–chitosan) groups over the 12 weeks. Both groups started with comparable baseline values (~ 1000 HU) and showed a decline by week 1. However, the Col/Cs group demonstrated a more robust recovery, with significantly higher CBMD values at week 12 compared to the control group (~ 1000 HU vs. ~950 HU; *P* < 0.0006). (Fig. 5B**).**

**Fig. 8:**
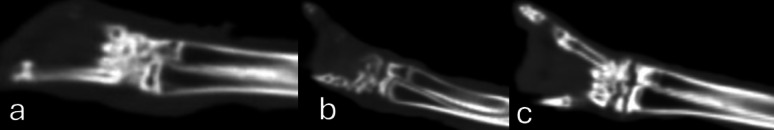
Coronal oblique reformatted CT images of the radio-carpal joint for the control group after intervention (**a**) after one week, (**b**) after 1.5 months, and (**c**) 3 months intervals showing minor degree of osseous bridging at 1.5- and 3-month intervals, respectively with a fusion ratio of about 34% at 3-month intervals

At baseline, ulnar bone mineral density (UBMD) was comparable between the control and collagen–chitosan (Col/Cs) scaffold groups, with both averaging approximately 780 HU. By week 1, a significant reduction in UBMD was observed in the control group compared to the Col/Cs group (*p* < 0.0001). Although partial recovery was noted in both groups from week 6 onward, the Col/Cs group consistently exhibited higher UBMD values throughout the 12-week postoperative period. **(**Fig. 8**).**

## Discussion

Arthrodesis stands out as a superior treatment option, delivering reliable results with minimal complications, predictable functional scores, and heightened patient satisfaction compared to total joint arthroplasty [15]. In guided bone tissue regeneration, clinicians frequently utilize biomaterials as barrier membranes to inhibit fibrous tissue infiltration into the wound site while promoting and directing the process of bone healing [16]. Bioactive polymer scaffolds are widely used in tissue engineering and regenerative medicine to support cell growth, proliferation, and differentiation [17]. These structures provide a 3D microenvironment that mimics the extracellular matrix (ECM), facilitating nutrient diffusion, waste removal, and cell migration [18]In this context, highly porous scaffolds are essential for supporting the natural cellular activities. Advances in biomaterials and fabrication techniques continue to improve their design for better cell growth and tissue regeneration [19].

Over the past thirty years, chitosan has been widely investigated as a naturally derived polymer with great potential for developing biomimetic scaffolds in bone tissue engineering. Thanks to its unique characteristics, various chitosan-based biomaterials such as microporous structures, fiber-reinforced forms, hydrogels, microspheres, and 3D-printed constructs have been engineered, each tailored to suit specific applications in tissue regeneration [20].

Marine-derived collagen has been increasingly combined with bio ceramics and other structurally robust materials to enhance its mechanical integrity and functional stability. it also enhances bone mineral density and stimulates osteoblastic activity, offering protective effects against bone degeneration [21]. In the context of bone tissue regeneration, these composite biomaterials facilitate new bone formation through the controlled release of bioactive ions, particularly calcium and phosphate, which play a central role in the mineralization process and the development of mature bone matrix [22].

The preparation of collagen/chitosan (COL/Cs) scaffolds using freeze-gelation and freeze-drying methods is a common approach to create porous scaffolds for tissue engineering and other biomedical applications [18]. The Col/Cs sample displayed a heterogeneous and interconnected porous microstructure with pore sizes over 100 microns and a reasonable thickness of pore walls. Indeed, numerous previous studies show that heterogeneous porous scaffolds are very useful for promoting the co-culture of several cell types, including fibroblasts and osteocytes, which are critical for the regeneration of bone and cartilage [23]. Furthermore, various cell types can be localized in different areas of the scaffold due to the variable hole sizes (up to 100 microns), which encourages their interaction and tissue development [24]. These findings confirmed that the COL/Cs scaffolds offer optimal microstructure features for cartilage tissue regeneration.

Compared to traditional approaches, the collagen–chitosan composite demonstrated favorable integration on CT imaging, safe for cells, showing no cytotoxicity, and supporting the growth and proliferation of bone cells strong antibacterial effects, reducing infection risk [25]and also addition of chitosan in collagen-based materials enhances the mechanical strength and stability, making them suitable for scaffolds [26].In contrast, bone grafts, whether autografts or allografts, pose challenges such as donor site morbidity and potential immunogenicity [27]. Synthetic materials like PMMA Poly (methyl methacrylate) and bioceramics may lack adequate biodegradability or osteoinductive properties [28]. Materials with poor mechanical properties (e.g., pure hydroxyapatite) are prone to fracture and are unsuitable for many orthopedic applications [29].

Multislice computed tomography (MSCT) stands as the gold standard for vividly capturing fractures, uncovering small bone fragments, and assessing articular surface involvement, especially when traditional X-rays fall short of providing precise results [30]. Moreover, the use of multislice computed tomography (MSCT) provides a powerful, non-invasive method for high-resolution monitoring of bone healing dynamics over time. MSCT enables quantitative assessment of bone regeneration through measurements such as Hounsfield unit (HU) values, cortical thickness, and fusion ratio, offering reliable insights into the progression of conditions like ankylosis or bone fusion [13, 31]. Studies have shown that MSCT-derived radiodensity measurements strongly correlate with histomorphometric data, validating the accuracy of imaging-based assessments for bone quality and healing [13, 32]This correlation means that MSCT can serve as a surrogate for more invasive histological evaluations, making it especially valuable in both experimental and translational orthopedic research [32] Also, CT allows for longitudinal, in vivo studies and repeated measurements, which are not possible with histology [33].

Cortical thickness is a critical morphological parameter influencing bone strength, as it directly correlates with the bone’s ability to withstand mechanical stress [34]. While increased cortical thickness contributes to enhanced bone strength, its effect is comparatively modest. Notably, the external diameter of long bones accounts for up to 55% of the variability observed in bone strength [35].

At one week post-surgery, both groups showed initial radial cortical thinning, resulting from localized inflammation and compromised periosteal blood supply, which stimulates osteoclastic bone resorption, marking an early catabolic phase that precedes osteoblastic activity and is characteristic of the initial stages of bone healing [36]. However, the Col/Cs group showed a continuous increase in cortical thickness throughout the 12 weeks, with significantly greater values compared to the control group at week 12 attributed to the synergistic osteoconductive and bioactive properties of the scaffold. Chitosan, a natural polysaccharide, enhances osteoblast adhesion, proliferation, and differentiation [37]. While collagen mimics the native bone extracellular matrix, providing a structural framework that supports cell migration and matrix deposition [38].

Bone mineral density (BMD) is a key indicator of bone strength and is widely used to assess fracture risk and diagnose osteoporosis, as it reflects the amount of mineral content in bone tissue [39]. There is a strong correlation between BMD and the mechanical strength of bone, particularly in cortical bone, where higher BMD generally predicts greater compressive strength and elastic modulus [40]. Interestingly, the Col/Cs group demonstrated a gradual increase in RBMD over the healing period, surpassing the control group by week 12. This late-phase enhancement of bone mineral density agreed with Su et al. [41]displaying that chitosan-collagen membranes and scaffolds support bone regeneration and contribute to increased bone density. The initial reduction in mineral density, followed by a significant increase, supports the hypothesis that scaffold implantation triggers a dynamic bone turnover process, an essential phase for successful scaffold incorporation and long-term bone consolidation.

Although a marked increase in intra-articular tissue mineral density (MD) was observed in the Col/Cs group at week 1 compared to baseline (*p* < 0.0001), this elevation does not reflect true mineralization. This early rise can be attributed to the hydrogel scaffold’s high-water content, as hydrogels typically contain a substantial amount of water, which can contribute to increased radiodensity on imaging [42].

Quantitative assessment of the joint space demonstrated a significant reduction in the Col/Cs group compared to the control group, indicating enhanced progressive osseous bridging, reflecting a more favorable osteoconductive environment. This outcome supports the hypothesis that the incorporation of collagen and chitosan synergistically enhances early extracellular matrix deposition, supports mesenchymal cell recruitment, and promotes endochondral ossification at the fusion site. These findings are in agreement with previous reports that highlight the role of natural polymers in modulating the local microenvironment to favor osteogenesis and structural joint integration during arthrodesis [43, 44].

## Conclusion

The result of the present study indicated that the collagen-chitosan group exhibited increased radial cortical thickness, enhanced bone mineral density, reduced joint space, and a higher fusion ratio compared to the control group. It provides an optimal osteoconductive environment, promoting early bone remodeling and increased. cortical thickness, improved bone mineral density, and accelerated osseous bridging.

### Study limitations

Although MSCT is used for the first time as a validating less invasive method of evaluation method it enables quantitative assessment of bone regeneration through Hounsfield unit (HU) values of bone mineral density, cortical thickness, and fusion ratio. However, detailed cellular and tissue-level events during types of bone fusion may need a histological analysis.

Further studies are warranted to evaluate long-term integration, mechanical strength, and potential translational applications in larger animal models or human clinical trials.

## Data Availability

No datasets were generated or analysed during the current study.

## Abbreviations

ANOVA: Analysis of Variance
BMD: Bone Mineral Density
CBMD: Carpal Bone Mineral Density
COL/Cs: Collagen/Chitosan
CT: Computed Tomography
HU: Hounsfield Units
IATMD: Intra-Articular Tissue Mineral Density
Kα: K-alpha radiation
MSCT: Multislice Computed Tomography
PMMA: Poly (methyl methacrylate)
PO: Post-Operative
RBMD: Radial Bone Mineral Density
ROI: Region of Interest
SD: Standard Deviation
SEM: Scanning Electron Microscopy
UBMD: Ulnar Bone Mineral Density
XRD: X-ray Diffraction
